# Temporal Regulation of Gene Expression in Post-Mitotic Cells is Revealed from a Synchronized Population of *C. elegans* Larvae

**DOI:** 10.17912/micropub.biology.000587

**Published:** 2022-06-10

**Authors:** Peter J. Roy

**Affiliations:** 1 Department of Molecular Genetics, University of Toronto, Toronto, ON, M5S 1A8, Canada; 2 Department of Pharmacology and Toxicology, University of Toronto, Toronto, ON, M5S 1A8, Canada; 3 The Donnelly Centre for Cellular and Biomolecular Research, University of Toronto, Toronto, ON, M5S 3E1, Canada

## Abstract

Unsupervised Uniform Manifold Approximation and Projection (UMAP) plots of single cell sequencing data from synchronized *Caenorhabditis elegans* larvae yield tissue-specific data clusters, some of which are plotted as elongated archipelagos. These archipelagos likely represent a single cell type. I show that the pharyngeal archipelagos express a myriad of asynchronous temporally regulated genes, which likely accounts for their elongated topology. With one archipelago, I show that there is a high correlation between a) the base pair distance between the binding sites of an archipelago-specific transcription factor (HLH-6) and the transcriptional start site of the targeted genes and b) the timing of peak gene expression of those genes that are expressed in an archipelago-specific manner. Despite the correlation being made with only four genes, it prompts the hypothesis that the physical distance between a transcription factor and the relevant transcription start site may be an important factor in determining the temporal onset of transcription and transcript abundance.

**Figure 1. Additional Temporal Regulation of Gene Expression in C. elegans Larvae is Revealed through the Intersection of Multiple mRNA Sequencing Datasets:**
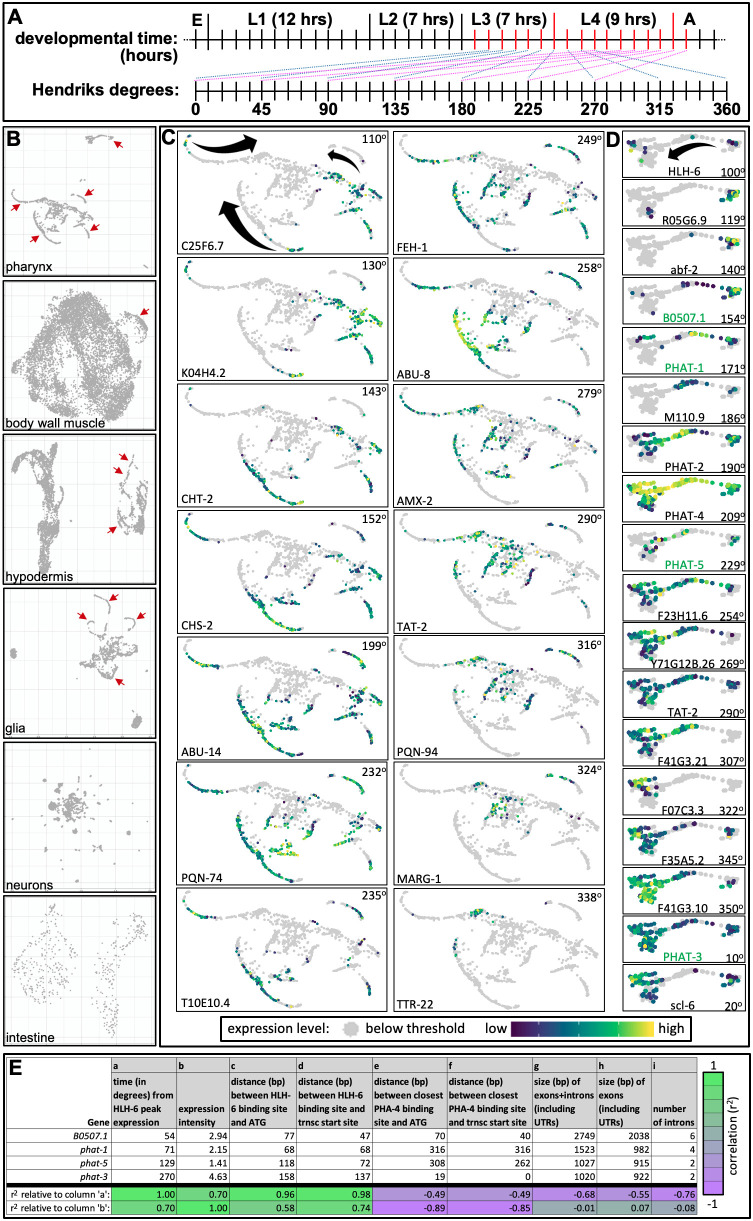
**A.** A schematic illustrating the translation of *C. elegans* developmental time at 25 ^o^ C (top timeline) to ‘degrees’ as articulated by Hendriks *et al.* , (2014) (bottom timeline). On the top timeline, red vertical lines indicate the approximate time points at which Hendriks *et al.* sequenced mRNAs from whole animals. Longer vertical lines denote the transition from one stage to the next. On the bottom timeline, the 360 degrees is the timeline of a repeating pattern of larval gene expression that has a periodicity of ~eight hours. For example, genes that peak in expression at one time point (blue diagonal lines) were observed to peak again ~eight hours later (pink diagonal lines). E, embryogenesis (~14 hours); A, adulthood. See Hendriks *et al.* , (2014) for more details. **B.** UMAP plots of six major post-mitotic tissue types. Red arrows highlight obvious archipelagos. **C.** UMAP plots from the pharynx, excluding the gland cells cluster (topmost cluster in the top panel in **‘B’** ) and two smaller clusters (on the bottom right of the top panel in **‘B’** ). Each of the 14 exemplar genes shown are enriched in pharynx gene expression (see (Kamal *et al.* , 2022)) and peak in expression at different times (indicated by the degree in the upper right-hand corner of each panel) that illustrate how expression progresses along each archipelago over time. The black arrows in the upper left panel indicate the flow of time. **D.** UMAP plots of genes expressed within the pharynx gland cells archipelago. Gene names in green are characterized HLH-6 targets and described in more detail in panel ‘ **D** ’. The description is the same as for **‘C’** . The scale of expression intensity is shown below ‘ **C’** and **‘D’** . All panels in **B-D** are unmanipulated screen shots from the VisCello server (see text for details). **E.** Four transcriptional targets of the HLH-6 and PHA-4 transcription (trnsc) factors and their associated properties. The source of the data in each column is as follows: a- (Hendriks *et al.* , 2014); b- (Cao *et al.* , 2017); c- (Smit *et al.* , 2008); d- (Smit *et al.* , 2008) and WormBase (WS284); e-(Smit *et al.* , 2008); f- (Smit *^et al.^* , 2008) and WormBase; g-i- WormBase. The bottom two rows show the correlation (r ^2^ ) between the indicated data columns as calculated in excel.

## Description

With the help of colleagues, I bioinformatically reconstructed a spatiotemporal map of pharyngeal cuticle development (Kamal *et al.* , 2022). I did this by intersecting several large-scale expression datasets. The reconstruction relied heavily on the Großhans group’s temporal expression dataset of *C. elegans* , whereby global mRNA expression (bulk RNAseq) was profiled every hour for 16 hours from the third larval stage animals to young adults (Hendriks *et al.* , 2014) (Figure 1A). In this initial study, it was found that 2718 genes oscillate in their expression level, with each particular gene peaking in expression at the same relative time at each larval stage, but at a different time than other oscillating genes. Given that oscillatory gene expression is cyclical from one stage to the next and that the duration of each larval stage is different, Großhans and colleagues compressed a full oscillation cycle into 360 degrees, with each hour equating roughly to 45 ^o^ and the time of peak expression for each gene corresponding to a particular degree within the cycle (Hendriks *et al.* , 2014).

Another key dataset used to build the spatiotemporal map was the *C. elegans* single cell sequencing (scRNAseq) dataset from synchronized second larval stage animals (Cao *et al.* , 2017). Packer and colleagues have provided an online ‘VisCello’ tool to visualize expression patterns of individual *C. elegans* genes on a global or tissue-specific scale (see https://cello.shinyapps.io/celegans_L2/ )(Packer *et al.* , 2019). What is immediately apparent with the tissue-specific UMAP plots of the scRNAseq data is how select cells from some tissues are plotted out along relatively long lines, which I call archipelagos for lack of a better term (red arrows in Figure 1B). Archipelagos manifest from tissues that are both mitotic (e.g., select cells within the hypodermis) and post-mitotic (e.g., the pharynx).

I examined the expression pattern of genes that I found to both oscillate in their expression level and are enriched in expression in the pharynx ((>1.5 fold enriched in the pharynx relative to all other tissues and at least 25 transcripts per 1 million reads) (Kamal *et al.* , 2022)) on the VisCello tool. I then arranged the resulting UMAP plots in order of the time in which they peak in expression. A clear spatial progression pattern emerges along the pharyngeal archipelagos (Figures 1C and 1D). Crude aspects of this temporal progression have been previously noted directly on the online VisCello output (input ‘pharynx’ sample, ‘UMAP-2D’ projection, and coloured by ‘cell type’).

I interpret the refined pattern of gene expression along the archipelagos illustrated in Figures 1C and 1D to mean that a single archipelago represents a single cell type that is changing in its expression profile gradually and meaningfully over developmental time. For example, the epithelial cells of the pharynx that contribute to pharyngeal cuticle synthesis and destruction during each larval stage form these archipelagos on the pharynx UMAPs (Kamal *et al.* , 2022). I infer that the cells within the archipelagos from other tissues are also playing a role in important temporally-regulated biological processes, although I cannot formally rule out other explanations such as changes in the animal’s size, architecture, or environment etc. Conversely, those cells that are not arranged into archipelagos may have less gene expression differences over developmental time (see body wall muscle and neuron UMAP plots for example). I expect that temporal programs of gene expression within the post-mitotic cells could be accurately predicted through computational analyses of the expression patterns on UMAP archipelagos alone without the need for global temporal RNA seq analyses in much the same way that pseudotime is inferred with complex mitotically proliferating systems (Liu *et al.* , 2021).

At a technical level, the observation suggests that the animals profiled by scRNAseq were not precisely synchronized, which is expected given the stochastic variability of rearing large numbers of synchronized animals. Indeed, Supplementary Figure 8 of the Cao *et al.* (2017) work explicitly argues this point. Alternatively or additionally, time may be manifested on UMAP archipelagos because there is biological variance to the regulation of temporal gene expression within a synchronized population.

Next, I inferred that temporally-oscillating transcription factors are likely driving the tissue-specific temporal regulation of gene expression. I therefore examined each of the cell types within the pharynx expression dataset for transcription factors that have been previously described in the literature as tissue or cell-specific. Jeb Gaudet’s group previously showed that the HLH-6 transcription factor drives gland-specific expression of a group of genes that includes the *phat* ShKT-domain-encoding paralogs (Ghai *et al.* , 2012; Smit *et al.* , 2008).

HLH-6 peaks in expression at 100 ^o^ on the oscillatory map (Figure 1D). Four genes that are expressed specifically in the gland cells and whose expression is HLH-6-dependent and whose upstream sequence has been shown to be bound by HLH-6 include *B0507.1* , *phat-1* , *phat-5* , and *phat-3* ; each of these genes peak in expression at 154 ^o^ , 171 ^o^ , 229 ^o^ and 10 ^o^ , respectively (Figure 1D). For each of these four genes, the HLH-6 binding site in the upstream sequence has been defined (see Supplemental Figure 1 in (Smit *et al.* , 2008)). Remarkably, there is a positive correlation between a) the distance of the HLH-6 binding site from the predicted initiator methionine ( *r ^2^* =0.96) or the predicted transcriptional start site ( *r ^2^* =0.98) and b) the onset of peak expression relative to the timing of HLH-6’s peak expression (Figure 1E). In other words, the closer the binding site of HLH-6 to the transcriptional start site, the sooner that transcript peaks in expression in this admittedly small sample size.

PHA-4 is a master transcription factor regulator of pharynx development (Mango *et al.* , 1994). The predicted PHA-4 binding sites of the four HLH-6-regulated genes have also been described (see Supplemental Figure 1 in (Smit *et al.* , 2008)). For each of the four genes, multiple PHA-4 binding sites are described (Smit *et al.* , 2008). Considering only the closest PHA-4 binding site, there is a strong negative correlation between the distance of the PHA-4 binding site from the predicted initiator methionine and transcript abundance ( *r ^2^* = -0.89) (Figure 1E). In other words, the closer the PHA-4 binding site, the more intensely-expressed an HLH-6-driven transcript is, again in this admittedly small sample size.

One obvious trivial explanation for the control of the timing of peak expression of HLH-6-regulated genes might be the length of the transcript, where longer transcripts might peak in expression later. However, there is an anticorrelation ( *r ^2^* = -0.68) between the timing of peak expression of the four HLH-6 targets and their transcript length, indicating that longer transcripts are not resulting in a delay of peak expression (Figure 1E).

I conclude by hypothesizing that the physical distance between a transcription factor and the relevant transcription start site may be an important factor in determining the temporal onset of transcription and transcript abundance. A variety of tests are conceivable, and a greater number of temporally regulated genes whose expression in the same cell is controlled by the same transcription factor are required to substantiate the hypothesis.

## Methods

The methodology needed to reproduce the observations are described in the main text and figure legend.

## Reagents

No reagents were used in this analysis.
